# Novel Modifications in Endodontic Bioceramics to Improve their Properties: A Narrative Review 

**DOI:** 10.30476/dentjods.2025.104239.2513

**Published:** 2025-12-01

**Authors:** Maryam Enteghad, Mahdi Zerafat, Saleh Hadian, Yasamin Ghahramani

**Affiliations:** 1 Postgraduate Student Dept. of Endodontics, School of Dentistry, Shiraz University of Medical Sciences, Shiraz, Iran.; 2 Dept. of Endodontics, School of Dentistry, Oral and Dental Disease Research Center, Shiraz University of Medical Sciences, Shiraz, Iran.

**Keywords:** Bioceramic, Biocompatibility, Discoloration, Mineral Trioxide Aggregate, Regeneration

## Abstract

Bioceramics (BCs) have gained significant popularity in various dental applications, including regenerative procedures, apexification, and root-end filling obturation, due to their remarkable characteristics, such as biocompatibility, sealing ability, and bioactivity. Despite their versatile applications, these materials possess few drawbacks, including extended setting time, discoloration, granular consistency, and complex handling. It is imperative to consider reformulating these materials in order to improve their properties and meet their special needs. Additionally, further research is needed to ensure their safety for clinical use. In recent decades, modifications in mineral trioxide aggregate (MTA) composition and properties have been a topic of research interest. This study aims to review recent advancements in modifying the chemical, mechanical, and biological properties of this versatile material and introduce some new commercial products to help practitioners select their required material wisely and assist researchers in planning future investigations.

## Introduction

The science of medicine and dentistry has undergone a revolution since bioceramics (BCs) were introduced and used in various fields such as orthopedic and maxillofacial prostheses [ 1 - 2 ], ocular implants [ 3 ], dental restorations and adhesives, air abrasions, enamel demineralization, and so on [ 4 ]. Subsequently, endodontic science and practice have advanced because of the unique properties of these materials, which have allowed practitioners to improve the prognosis of hopeless teeth.

Ceramics are a class of non-metallic inorganic materials produced by applying high temperatures to minerals [ 5 ]. Ceramics and BCs differ in terms of their biocompatibility, antimicrobial activity, and improved sealing ability that allow them to be employed in the human body. They can be placed and function as the damaged tissue and stimulate the formation of healthy new tissue [ 6 ]. BCs are categorized in different ways. Based on their composition, they are classified as zirconia, alumina, glass ceramics, bioactive glass, calcium silicates, calcium phosphates, hydroxyapatite, and radiotherapy glasses. Depending on their reactivity with surrounding tissue, we classify them as either bioinert (causing little reaction with the tissue around it such as alumina, zirconia), bioactive (eliciting biological responses, such as bioactive glasses, hydroxyapatite, calcium silicates), or biodegradable (can be replaced, reabsorbed, or integrated into tissue such as bioactive glasses, tricalcium phosphate)[ 7-9 ].

Some BCs containing alumina (Al2O3) have a long lifespan and can be employed in pacemakers, ocular prostheses, and middle ear ossicles. Vitreous carbon is also used as a cardiac valve replacement due to its compatibility with blood [ 10 ]. Dental prostheses and composites frequently contain zirconia and aluminosilicate. Also, BCs containing hydroxyapatite and calcium phosphate are employed in orthopedic and maxillofacial applications because of their resemblance to minerals in bone [ 11 ].

There are two broad categories for the BCs used in endodontic procedures: first, calcium silicate-based cements, and second, not based on calcium silicate (such as calcium phosphates/ tricalcium phosphate/ hydroxyapatite/ calcium aluminate) [ 12 - 13 ].

The first commercial mineral trioxide aggregate (MTA) (ProRoot® vc® MTA, Dentsply Tulsa Dental, USA) is claimed to be comparable to Portland cement in terms of pH, biocompatibility, solubility [ 14 - 15 ], seal ability [ 16 - 17 ], antibacterial capacity, and chemical composition (apart from bismuth oxide), but to contain less iron-3 (Fe3) and aluminum [ 18 ]. Other distinctions between the two materials include the particle size, which is smaller and more uniform in MTA [ 19 ], and the radiopacity, which is more significant in MTA [ 20 ]. The crystallization process in MTA is more organized than in Portland cement, which leads to better bioactivity in MTA [ 12 ].

The goal of this review is to evaluate the literature on modifications in MTA composition and properties and review prominent features of new commercial products using an evidence-based approach to help practitioners select materials as their necessities and investigators to further research.

Although the first application of Portland cement described in dentistry literature was documented in the 1880s [ 10 ], a century later, Dr. Torabinejad introduced MTA, which led to a revolution in endodontic practice [ 21 ]. The initial MTA invented was composed of the same di- and tricalcium silicate main parts as Portland cement, with around 20% bismuth oxide added as a radiopacifier [ 21 ]. Hydration is the mechanism by which it sets, creating hydrated calcium silicate and calcium hydroxide, which in turn interact with phosphate ions in the body to produce hydroxyapatite. So it is called osteoconductive and osteoinductive [ 6 ].

When comparing white and gray MTA of the same commercial brand, similar biocompatibility [ 22 ], microleakage [ 23 ], antibacterial action [ 24 ], marginal adaption [ 25 ], and biological response are demonstrated in different applications [ 26 ]; however, the white MTA has finer particles and less iron oxide [ 27 ]. White MTA's enhanced surface hardness makes it a viable option in situations involving blood or serum contamination [ 28 ]. In clinical application, gray MTA showed a higher proportion of dentine bridge formation than white MTA [ 29 ]. So, it would be vulnerable to look into how the composition affects the material’s properties and performance in clinical applications.

## Search Strategy

Four independent reviewers conducted a comprehensive literature search to find related English articles in PubMed, Web of Science, Scopus, and Cochrane Library databases, between January 1, 2005, and February 15, 2024. We used the following search method to discover relevant studies: (bioceramics OR hydraulic cements OR calcium silicate-based cements) AND (root canal OR endodontics OR root canal treatment).

### Inclusion and Exclusion criteria

The review covered clinical and laboratory studies that looked into at least one of the endodontic BCs’ properties. Also, the reference list of included studies and previously published books and reviews were searched. The studies performed on artificial resin teeth were excluded.

## Results

According to the mentioned search strategy, 71 articles were chosen for review and article writing. Among them, 19 articles were related to the setting reaction, 12 articles focused on the mechanical properties, 17 articles evaluated tooth discoloration, 16 articles were about their biocompatibility and regenerative potential and 7 articles assessed different aspects of BCs’ properties.

## Discussion

**Figure 1:**
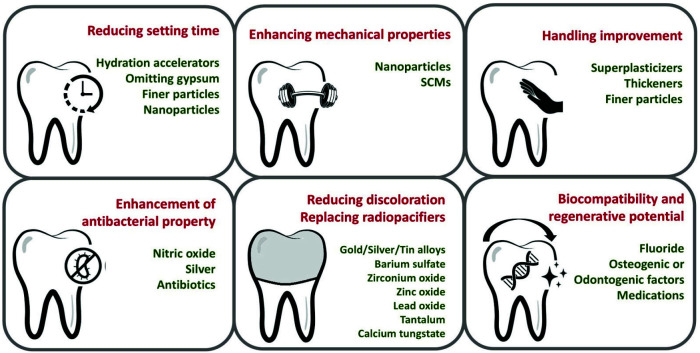
An overview of six primary areas of advancement achieved in novel bioceramics to overcome the drawbacks associated with the conventional formulation of mineral trioxide aggregate (MTA), such as prolonged setting time, discoloration, and difficult manipulation, among others

The properties of MTA can be affected by different environmental factors, such as blood, saliva, dental restoration, and tissue fluids [ 30 ]. The issues of graininess and poor consistency, long setting time, low compressive strength immediately after mixing, discoloration, and difficult handling have prompted researchers to look for solutions [ 31 ]. This article provides an over- view of the main areas of advancement achieved in novel BCs to overcome the drawbacks associated with the conventional formulation of MTA (Figure 1).

### Enhancing setting time Gypsum omission

Following the introduction of the first commercial MTA ProRoot, MTA Angelus (Angelus, Londrina, Brazil; Clinician’s Choice, New Milford, CT) was released with a brilliantly faster setting time reaction (50 min) compared to ProRoot (over two hours) by omitting gypsum (calcium sulfate), which is the retarder of the reaction. However, a superplasticizing component may be required to improve the cement’s workability. Additionally, MTA Angelus contains different amounts of dicalcium and tricalcium silicate and less bismuth oxide than ProRoot MTA, resulting in less opacity [ 32 ]. Proto A (Aalborg White, Aalborg, Denmark) is another cement that shares the same basic elements as MTA but without gypsum, resulting in a quicker setting. Also, the product’s water bottle contains some superplasticizers, helping to improve its workability [ 33 ].

### Calcium aluminate

The admixture of calcium aluminate to Portland cement speeds up the setting time, however, it weakens the material’s compressive strength. The addition of calcium sulfate can help enhance compressive strength by creating calcium sulfoaluminate [ 34 ].

### Hydration accelerators

Several investigations have been carried out to look into the effects of hydration accelerators, including calcium chloride (CaCl2), calcium formate, calcium nitrate, and calcium lactate gluconate, when combined with MTA [ 35 - 36 ]. They have consistently demonstrated a faster setting time, improved sealing ability, and better handling characteristics without detrimental effects on biocompatibility. However, it should be noted that adding these accelerators was associated with a decrease in compressive strength [ 35 ]. Furthermore, a study reported that the inclusion of CaCl2 in MTA, when used as a pulp capping biomaterial, has a negative impact on calcific bridge formation and promotes inflammation responses [ 36 ]. Different results could be attributed to the material’s concentration. Examples of BC materials that use CaCl2 as hydration accelerator include, but are not limited to, Biodentine (Septodont, Saint-Maur-Fosses Codex, France), Endo-CPM sealer (EGEO SRL, Buenos Aires, Argentina), MTA-Caps (Acteon, Merignac, France), calcium enriched mixture (CEM) cement (BioniqueDent, Tehran, Iran), and RetroMTA (Meta Biomed Co, Ltd, Seoul, Korea) [ 37 ].

### Polyphenolic compounds

Also, in a study conducted in 2021, it was observed that the use of a polyphenolic compound, such as tannic acid in MTA cement, resulted in a quicker setting time and superior surface and bulk properties by incorporating between the cement grains, leading to a reduction in grain size and the formation of robust intramolecular hydrogen and ionic bonds [ 38 ].

### Finer particles

Another approach that has been employed in a novel MTA (MTA Plus; Avalon Biomed Inc., Bradenton, FL, USA) is to use a composition similar to MTA but with smaller particles that give the material a higher specific surface area and accelerate the rate of setting reaction, but it does not necessarily shorten the final setting time [ 39 ]. On the other hand, nano fast cement (NFC, Sanat Avaran Vista, Iran) is a new product with a final setting time of only 15 minutes. It has comparable discoloration, biocompatibility, sealing ability, and antibacterial effect to Proroot white mineral trioxide aggregate (WMTA) and contains zirconium oxide as a radiopacifier [ 40 - 42 ].

### Addition of nanoparticles

Furthermore, there has been interest recently in combining other nanoparticles with MTA to address its inadequacies. A recent study demonstrated that adding silica nanoparticles accelerated the hydration process, shortened the setting time, and improved the material’s compressive and flexural strengths [ 43 ]. Nevertheless, a further study assessing the impact of including calcium carbonate nanoparticulate into MTA revealed an accelerated setting time, accompanied by a reduction in compressive strength and an increase in solubility [ 44 ].

### Gelatin/ Chitosan

**Figure 2:**
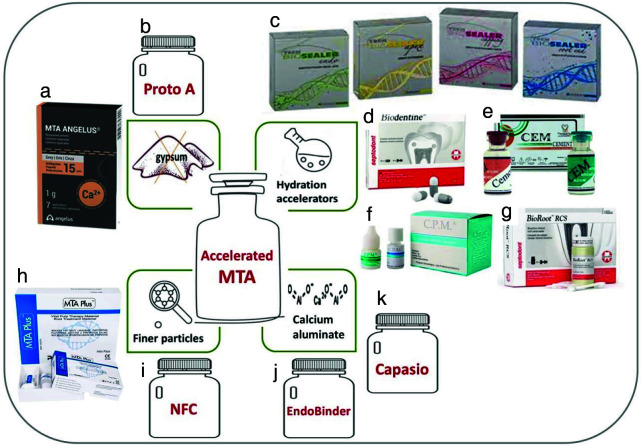
A summary of the approaches to accelerate mineral trioxide aggregate (MTA) setting time and some related commercial products. The initial attempt to expedite MTA's setting time involved the exclusion of gypsum. Additional alterations involve the incorporation of hydration accelerators, finer particles, and calcium aluminate. **a:** MTA Angelus, **b:** Proto A, **c:** Tech Biosealer, **d:** Biodentine **e:** CEM- Cement, **f:** Endo-CPM sealer, **g:** BioRoot RCS, **h:** MTA Plus, **i:** Nano Fast Cement (NFC), **j:** EndoBinder, **k:** Capasio

On the other hand, one drawback of MTA is its tendency to wash out, which relates to the susceptibility of freshly mixed cement to breakdown upon exposure to blood or other fluids due to its prolonged setting time. Previous studies have provided evidence that adding gelatin or carboxymethyl chitosan to calcium silicate- based cement enhances its resistance to washout [ 45 - 46 ] (Figure 2).

### Enhancing mechanical properties Nanoparticles

Several attempts have been undertaken to improve the characteristics of MTA, including the application of nanoparticles, as previously mentioned. Although the inclusion of calcium formate, calcium chloride, calcium carbonate, and di-sodium hydrogen orthophosphate nanoparticles decreased the compressive strength of MTA [ 47 - 48 ], the incorporation of hydroxyapatite and zinc oxide nanoparticles into MTA did not have any detrimental effect on its strength. Instead, these nanoparticles can potentially improve other properties of MTA, such as biocompatibility and antimicrobial properties in experimental studies [ 49 - 50 ]. An experimental study conducted in 2021 revealed that the addition of graphenenanoplatelets to Angelus MTA leads to an elevation of the carbon ratio without any change in crystal structure, resulting in an enhancement in hard tissue formation potential, as well as improvements in microhardness and resilience under permanent restorations [ 51 ].

### Supplementary cementitious materials (SCMs)

Another technique to alter the mechanical properties of hygroscopic dental cements is the addition of SCMs, which are mineral admixtures that do not undergo a chemical reaction with water but can react with aqueous calcium hydroxide. This process leads to a lower porosity and higher strength of the final cement. SCMs typically contain a high amount of silica and include fly ash, slag, and natural pozzolans [ 52 ]. Using this approach, Endocem MTA (Maruchi, Wonju, Korea), a novel MTA-derived pozzolan cement, exhibits a faster setting time and improved washout resistance when compared to ProRoot MTA, while maintaining similar biocompatibility and handling characteristics [ 53 ].

### Handling improvement

Manipulating MTA is difficult because of its dry and granular consistency. As the mixture dries, it loses cohesiveness, which makes it more challenging to handle at the surgical site [ 54 ]. Another disadvantage of using MTA as a root canal filler is that it can be difficult to obturate, particularly in curved canals .

### Superplasticizers

The inclusion of rheological modifiers, such as superplasticizers and thickening agents, can help to improve handling properties to meet specific needs. When superplasticizer additives are ccc used, the ratio of water to cement goes down. This makes the material easier to work with and gives it better mechanical properties [ 57 ]. As an illustration, the polycarboxylate plasticizer in Bio- dentine reduces the material’s viscosity and setting time [ 58 ]. It also helps to improve adhesion to dentine and thereby increases the resistance of the material to dislodging forces [ 59 ]. This material is particularly designed to serve as a “dentin replacement” material. So, it does not require a two-step restoration procedure as in the case of MTA [ 12 ].

### Thickening agents

Thickeners can be added to the water bottle of MTA to modify the material’s viscosity, helping to produce a paste (for endodontic sealers) or a putty (for restorative purposes). For example, the addition of propylene to MTA Angelus makes a paste that is easier to manipulate. Notably, the ratio of above 20% propylene glycol interferes with setting time [ 54 ].

### Premixed formulations

The novel premixed and ready-to-use BCs have the benefit of precise and homogenous consistency that would simplify their handling by reducing material and time waste [ 60 ]. They have the same basic chemical composition as the other BC products and may contain poly- ethene glycol, fillers, and thickening agents . They are hydrophilic in nature and require moisture from the surrounding tissues to set. Based on their consistency, they are available in the form of syringable sealers or putty-form cements. Some of the commercially premixed BCs include, but are not limited to, iRoot BP (Innovative Bioceramics, Vancouver, Canada), Endo Sequence root repair (Brasseler USA, Savannah, GA), TotalFill (FKG Dentaire SA, Switzerland), Neosealer and Neoputty (NuSmile Avalon Biomed, Bradenton, FL, USA) [ 63-64 ].

### Particle size

Another method to modify handling is changing the particle size of the components, which has a direct impact on the flow behavior of the material [ 65 ]. Recently, a new MTA-based material, MTA HP (high plasticity) (Angelus, Londrina, Brazil), has been launched into the market. One notable distinction between this novel material and the previous formulation of white MTA Angelus is substituting distilled water with a liquid containing water and an organic plasticizer that facilitates the material’s manipulation while being inserted into the repair site. The other difference between the two materials is the finer particles in MTA HP. The consistency obtained after mixing is more like a modeling mass, not sandy [ 66-67 ].

Another new material, the MTA Flow (Ultradent Products Inc., South Jordan, UT), is composed of a very small particles of tri- and dicalcium silicate and a liquid vehicle of a silicone-based gel that presents a higher degree of plasticity. This combination produces a creamy and homogenous material after mixing. Different consistencies depending on the liquid-to-powder ratio enable its application in diverse procedures [ 68-69 ].

### Enhancement of the Antibacterial Properties

ProRoot MTA exhibits an antibacterial effect against some facultative bacteria, which attributed to the material’s high pH and active calcium hydroxide release [ 70 ].

### Antimicrobial agents

Several studies have assessed the efficacy of antimicrobial agents such as chlorhexidine, sodium hypochlorite, iodoform, and silver zeolite when combined with MTA. Although the incorporation of 0.12% chlorhexidine gluconate into MTA enhanced the antimicrobial properties for at least 48 hours, it decreased the material’s compressive strength, prevented its setting process, and caused cell apoptosis . The addition of 3% sodium hypochlorite led to a decrease in cell viability in freshly mixed MTA [ 73 ]. Also, no antibacterial effect was observed in combination with iodoform and MTA. Nevertheless, the addition of antimicrobial agents is not usually recommended due to their possible discoloration potential and cytotoxicity [ 74 ].

### Nitric oxide

Also, in a recent study on the biocompatibility and antimicrobial properties of the mixture of MTA and a nitric oxide-releasing compound, they found that the mixture was more effective than MTA alone against Enterococcus faecalis and Porphyromonas endodontalis. Also, both MTA and MTA- nitric oxide compounds could stimulate cell proliferation and enhance the production of alkaline phosphatase, which contributed to the regenerative potential of the pulp tissue [ 75 ].

### Silver nanoparticles

Additionally, a study investigating the impact of incorporating silver particles [ 76 ] into MTA-based endodontic sealers presented enhanced antibiofilm capability and calcium release while not significantly affecting other characteristics [ 77-78 ].

### Antibiotics

Lastly, research has been done to assess antibacterial qualities of MTA when used in conjunction with antibiotics as a drug delivery agent. A study evaluating the incorporation of amoxicillin-loaded microspheres into MTA showed that although it lengthened the material’s setting time; the controlled release of antibiotics could be an alternative way to deliver antibiotics locally to the infected tooth [ 79 ]. The addition of metronidazole to MTA is also reported to enhance the antimicrobial properties of the modified MTA with no alteration in its physical properties [ 80 ].

### Reducing the Discoloration

Although gray MTA causes noticeable crown discoloration after one month, in white MTA, it becomes distinguishable after three months [ 37 ]. Bismuth oxide, the radiopacifying agent, is linked to tooth discoloration in both gray and white MTA. When bismuth oxide is exposed to potent oxidizing agents such as sodium hypochlorite or amino acids, it undergoes a state of instability. Moreover, the presence of arsenic and iron in several BC cement compositions can cause discoloration [ 37 ].

**Figure 3:**
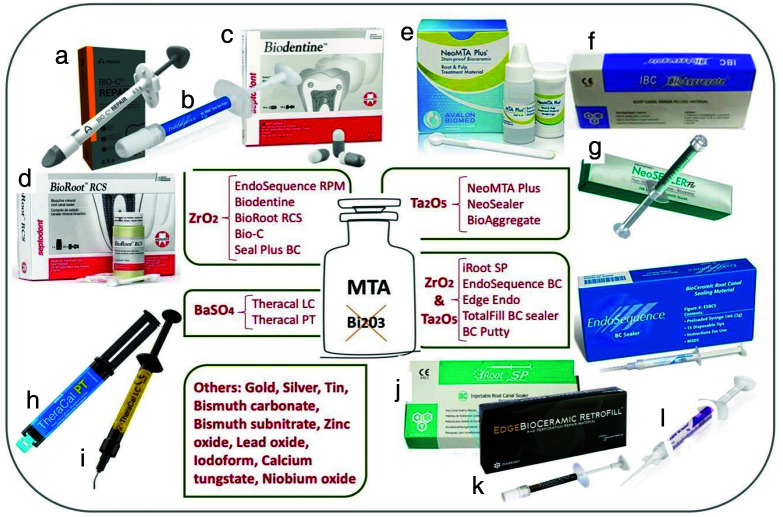
An overview of substituting bismuth oxide with other nontoxic, inert, and colorless substances, along with the introduction of some related non-staining products, **a:** Bio-C Repair, **b:** EndoSequence RPM, **c:** Biodentine, **d:** BioRoot RCS, **e:** NeoMTA Plus, **f:** Bio- Aggregate, **g:** Neosealer, **h:** TheraCal PT, **i:** TheraCal LC, **j:** iRoot SP, **k:** Edge Endo Bioceramic Retrofill, **l:** TotalFill BC sealer

In recent BCs, bismuth oxide has been replaced with other non-toxic and colorless alternatives, including barium sulfate, zirconium oxide, zinc oxide, tantalum oxide and calcium tungstate, as shown in Figure 3 [ 81-82 ].

### Elimination of the Heavy Metals

Based on ISO 9917 guidelines, both Portland cement and MTA exhibit a minimal amount of arsenic and lead release, which is much less than the toxic dose in endodontic procedures [ 83 ].

MTA Bio (Angelus, Londrina, PR, Brazil), a water- based cement, is claimed to be synthesized in a laboratory under strictly monitored conditions to prevent the occurrence of arsenic and lead contaminants. It is reported to have a shorter setting time, low cytotoxicity, and comparable sealing ability [ 84 ].

### Light-Curable MTA

The light-curing resin-modified MTA systems mostly contain functional groups that are capable of chelating calcium ions, which leads to an alkalinizing pH and apatite formation. However, in the context of clinical procedures, a moderate chronic inflammatory response was observed 30 days after application as a pulp capping agent, which was more intense than with MTA Angelus, with no calcification or bridge formation [ 85 ] TheraCal LC (Bisco Inc, Schaumburg, IL) is a light- curable tricalcium silicate pulp capping agent. Its ability to release calcium is controversial due to its restricted fluid availability and limited material hydration. Also, its extracts were found to be cytotoxic when they came into contact with pulp cells [ 37 ].

### Enhancement of Radiopacity

Although radiopacifiers do not act as reagents in the hydration setting reaction, they can slow it down and change the physical properties of the cement [ 86 ].

### Bismuth oxide

Many dental materials, including root canal sealers and MTA, utilize bismuth oxide as a radiopacifier, enabling their distinction on a radiograph. Despite its high radiopacity, studies have reported that bismuth oxide in MTA does not present inert properties. In short-term culture, it exhibited high cytotoxicity and caused cell death. Also, it is associated with tooth discoloration, as previously mentioned [ 21 , 87 ]. In several studies, bismuth oxide was substituted with other inert, nontoxic, and colorless compounds including gold powder, silver/tin alloys, bismuth carbonate, bismuth subnitrate, barium sulfate, zirconium oxide, zinc oxide, lead oxide, iodoform, tantalum, and calcium tungstate [ 56, 81 ].

### Gold powder

The gold particles present a good radiopacity [ 88 ] when imparted to MTA. They are completely inert and do not leach away in solution; the main drawbacks are associated with the price and the resulting color of the cement caused by the presence of gold [ 81 ].

### Zirconium oxide

When zirconium oxide powder was added to Portland cement as a radiopacifier, the characteristics of the material resembled those of MTA, with no noticeable drop in compressive strength [ 82 ].

### Zirconium-doped Bi2O3

Several studies have suggested that the negative properties of Bi2O3 radiopacifier can be reduced by different metallic ion doping. A study by Peng *et al*. [ 89 ] demonstrated that the addition of Zirconium-doped Bi2O3 radiopacifier into MTA caused a better radiopacity, faster setting times, and enhanced mechanical strength. However, the study did not assess its possible influence on tooth discoloration, and this specific area should be the focus of future research [ 90 ].

### Improving biocompatibility and regenerative potential

Finally, the bioinductivity and biocompatibility of BCs are the most striking features that make them suited for regenerative endodontics, perforation repair, and vital pulp therapy. They have the capacity to promote the proliferation and differentiation of stem cells. Based on a systematic analysis, BC materials were found to share similar biological features to MTA, such as minimal cytotoxicity, cell adhesion and proliferation, and low inflammatory cytokine expression [ 91 ].

On the other hand, any changes made to the composition of these products should be examined in terms of their biocompatibility and regeneration qualities [ 92 ]. For example, although some hydration accelerators reduce the setting time of MTA, they can affect other properties that should be mentioned. Studies showed that the pulp reaction caused by MTA combined with 10% calcium chloride, 0.1% citric acid, or 15% disodium hydrogen phosphate was not noticeable; however, seven days following replantation, MTA mixed with 0.43% calcium lactate gluconate elicited moderate-to-severe inflammation [ 93-94 ].

### Fluoride

Several studies looking into the addition of fluoride to the MTA have shown that it enhances the material’s bioactivity, calcium release, and fluorapatite formation in phosphorus-rich environments. Fluoride-doped MTA is an innovative sealer incorporating sodium fluoride and has superior retention and expansion properties while decreasing microhardness [ 32 ]. Furthermore, another study assessing MTA containing calcium fluoride (CaF2) showed increased human dental pulp cell proliferation and gene expression linked to mineralization without compromising the material’s physical properties [ 95-96 ].

### Odontoblastic factors

Moreover, recent studies have demonstrated that a combination of MTA with a potent odontogenic protein can accelerate the differentiation of human dental pulp cells more quickly than MTA alone. According to a recent study, MTA plus combined with either parathyroid hormone-related protein or osteostatin has a synergistic effect on promoting mineralization and differentiation of osteogenic and odontogenic tissue [ 97 ].

### Medications

Finally, a recent area of dental research is exploring the feasibility of incorporating pharmaceuticals into MTA. A study examining the apical plugs of MTA mixed with triple antibiotic powder found that this modified material released more calcium ions, which accelerated the apexification process [ 98 ]. Also, a study conducted to evaluate how adding a vasodilating drug like iloprost to MTA affects its biological properties showed that MTA-iloprost considerably increased the level of bone sialoprotein, osteocalcin, and osteopontin expression compared to MTA, thereby enhancing the viability and osteogenic potential of human stem cells [ 99 ]. Furthermore, metformin has been reported to stimulate odontoblastic differentiation and root development [ 100 ]. It has been demonstrated that the addition of metformin to calcium phosphate cements can promote cell differentiation and induce mineralized tissue formation, offering a promising potential in regenerative field [ 101 - 102 ]. 

## Conclusion

BCs are among the most widely used materials for various clinical endodontic applications. While BCs are used in radicular part of the tooth for apexification, it is imperative to choose a material with adequate biocompatibility and regenerative potential. Mechanical strength and rapid setting time are imperative when the restoration must be placed immediately after the BC application. In endodontic periapical surgery, the applied BCs must have a proper handling and resistance to washout. So, it is reasonable to expect various characteristics in commercial products to satisfy any particular requirement. Most recent BC formulations contain additives to enhance their properties. Finally, we must acknowledge that these new innovative biomaterials have revolutionized the primary application areas of BCs in endodontics.
